# Evaluating the Cardioprotective Effects of Melatonin in Non-metastatic Breast Cancer Patients Receiving Doxorubicin Plus Cyclophosphamide: A Triple-Blind, Placebo-Controlled Randomized Controlled Trial

**DOI:** 10.5812/ijpr-162962

**Published:** 2025-10-20

**Authors:** Seyyed Mohammad Mousavinia, Farnoosh Larti, Hossein Ranjbar, Marzieh Lashkari, Kamran Roudini, Amir Hossein Emami, Mohsen Esfandbod, Zahra Jahangard-Rafsanjani

**Affiliations:** 1 School of Pharmacy, Tehran University of Medical Sciences, Tehran, Iran; 2 Department of Cardiology, Imam Khomeini Hospital Complex, Tehran University of Medical Sciences, Tehran, Iran; 3 Department of Internal Medicine, Cancer Institute, Imam Khomeini Hospital Complex, Tehran University of Medical Sciences, Tehran, Iran; 4 Radiation Oncology Research Center, Cancer Institute, Tehran University of Medical Sciences, Tehran, Iran; 5 Research Center for Rational Use of Drugs, Tehran University of Medical Sciences, Tehran, Iran

**Keywords:** Breast Cancer, Melatonin, Cardiotoxicity, Doxorubicin, Echocardiography, Global Longitudinal Strain

## Abstract

**Background:**

Doxorubicin, one of the most widely used chemotherapy drugs, has several side effects, including cardiotoxicity.

**Objectives:**

We investigated the effect of melatonin on doxorubicin-induced cardiotoxicity in breast cancer patients treated with a regimen of doxorubicin plus cyclophosphamide (AC).

**Methods:**

This is a triple-blind, placebo-controlled, randomized clinical trial conducted at the Cancer Institute of Imam Khomeini Hospital, Tehran University of Medical Sciences. Using the block randomization method, 63 breast cancer patients participated in the study and were randomly divided into two groups of 32 and 31, receiving melatonin (10 mg) and a placebo, respectively. The chemotherapy regimen for these patients included doxorubicin 60 mg/m^2^. Melatonin or placebo was started concurrently with doxorubicin at bedtime in the first cycle of chemotherapy and continued until one week after the end of the last cycle of chemotherapy. Echocardiography was performed before initiation and one week after the last chemotherapy session. Also, the cardiac troponin I (cTnI) and creatine kinase-myoglobin binding (CK-MB) levels were measured upon study recruitment, one week after the second and fourth chemotherapy sessions.

**Results:**

The echocardiography showed that after the intervention, the left ventricular ejection fraction (LVEF) was higher in the melatonin group than in the placebo group, but it was insignificant. Meanwhile, the average global longitudinal strain (GLS) was significantly higher in the melatonin group than in the placebo group at the end of the study. The cTnI and CK-MB biomarker levels were lower in the melatonin group compared to the placebo group. These changes were significant for cTnI but not for CK-MB.

**Conclusions:**

Melatonin may be effective in the prevention of doxorubicin-induced cardiotoxicity based on the improvement in GLS and biomarker levels.

## 1. Background

Anthracyclines are effective chemotherapy drugs in treating various cancers, including breast, lung, testicular, ovarian, thyroid, and hematological malignancies (1). Cardiotoxicity represents one of the most significant dose-limiting toxicities associated with anthracycline therapy, wherein oxidative stress has been identified as the predominant mechanistic pathway underlying this adverse effect (2-4). The prevalence of anthracycline-induced congestive heart failure (HF) depends on the drug dose, but on average, it has been reported to be between 4.5% and 26% (5). According to the 2022 ESC guidelines, cancer therapy-related cardiac dysfunction (CTRCD) patients can be divided into symptomatic and asymptomatic groups. Each group is categorized into mild to severe depending on the severity of symptoms, echocardiography results, and level of cardiac biomarkers (6).

Anthracycline-induced cardiomyopathy limits the treatment options. Diagnosing subclinical cardiac involvement is essential and will lead to prompt recognition, treatment, and accelerated HF recovery (7). Echocardiographic measurements of left ventricular ejection fraction (LVEF) and global longitudinal strain (GLS) are commonly used to detect overt and subclinical cardiac diseases in chemotherapy patients (8). Meanwhile, increased cardiac biomarkers after chemotherapy can be a significant risk factor and predictive factor for cardiotoxicity in the future (9). Various methods have been used to reduce cardiac toxicity caused by anthracyclines (10, 11), and many drugs and supplements, including carvedilol, spironolactone, ACE inhibitors, dexrazoxane, black seed oil, and many other compounds, have been studied for this purpose. Nevertheless, none of them have been recommended as a definitive solution for preventing or treating this condition (3, 11-14).

Melatonin (N-acetyl-5-methoxytryptamine) is a hormone released endogenously from the pineal gland and effectively regulates the circadian rhythm. However, it has been shown that melatonin has antioxidant, anti-inflammatory, antitumor, and cardioprotective effects (7, 15, 16). It also improves Body Mass Index (BMI) and modulates the immune system. Evidence has shown that melatonin may also play a role in pain relief and increase the effectiveness of chemotherapy (7, 15-17).

## 2. Objectives

Considering the cardiotoxicity mechanism of anthracyclines, which is through the production of free radicals, and the antioxidant, anti-inflammatory, and anti-apoptotic effects of melatonin (18), we decided to investigate melatonin's efficacy in the prevention of doxorubicin-induced cardiotoxicity. In many animal studies, melatonin's effectiveness in preventing cardiotoxicity caused by anthracyclines has been proven (18, 19), while there is still no human study. An animal study also demonstrated that antioxidants like green tea extract mitigate doxorubicin-induced cardiac injury by reducing oxidative stress (20).

The primary outcome was defined as the development of symptomatic HF or subclinical deterioration in myocardial function and deformation, as determined by LVEF and GLS. Changes in cardiac biomarkers, including cardiac troponin I (cTnI) and creatine kinase-myoglobin binding (CK-MB), were evaluated as secondary outcomes.

## 3. Methods

### 3.1. Study Type

This study was conducted as a triple-blind, placebo-controlled randomized clinical trial at the Cancer Institute Center of Imam Khomeini Hospital Complex, Tehran University of Medical Sciences, with registration reference IRCT20211109053025N1 approved by the Iranian registry of clinical trials. Additionally, it was approved by the Institute of Pharmaceutical Sciences of Tehran University of Medical Sciences with the code of ethics IR.TUMS.TIPS.REC.1400.195.

### 3.2. Study Population

Patients at least 18 years of age, newly diagnosed with nonmetastatic breast cancer, who were scheduled to receive adjuvant therapy, including doxorubicin plus cyclophosphamide (AC), were considered eligible. They should have normal liver and renal function. Patients were included in the study after reading and signing the informed consent form. Patients with baseline LVEF less than 50%, a history of melatonin use, ACE inhibitors, ARBs, and beta-blockers in the past three months, or patients undergoing any type of HF treatment (such as ARNi or CCB) were excluded from the study. Other exclusion criteria were a history of cardiovascular diseases such as hypertension, coronary artery diseases, cardiomyopathy, and arterial fibrillation, chest radiotherapy, and taking an antioxidant drug in the past month, including vitamin E, C, and N-acetyl cysteine. The remaining exclusion criteria were concurrent participation in other clinical studies and known sensitivity to any substances used.

After obtaining informed consent, the eligible patients were randomly divided into placebo and treatment arms (melatonin 10 mg) using the block randomization method. Randomization was done using a computer-generated randomization schedule (a block size of 4) created by the supervisor of the research. Boxes containing melatonin or placebo tablets with a similar appearance were numbered based on the randomization list and delivered to each participant based on their entry number. Patients, investigators, and statisticians were all blinded regarding the study recruitment arms. All melatonin and placebo pills were produced similarly by Jalinous Pharmaceutical Company, Tehran, Iran. The chemotherapy regimen for these patients included an AC regimen with four cycles at 2 or 3-week intervals, and the dose of doxorubicin was 60 mg/m^2^. Melatonin or placebo tablets were started on the same day as the doxorubicin infusion in the first cycle of chemotherapy with a dose of 10 mg at bedtime and continued for one week after the completion of the last course of chemotherapy. To increase patient adherence, the exact number of tablets was given to the patients in each chemotherapy session. Patient characteristics, including age, weight, underlying diseases, and drug history, were recorded. To monitor adherence, patients were assessed at each chemotherapy visit and asked about medication use. Moreover, medications were not dispensed all at once; the subsequent package was provided only after the completion of the previous one. The occurrence of side effects caused by melatonin (including daytime sleepiness, dizziness, headache, decreased concentration in doing tasks, and nightmares) was recorded periodically during the study. Adverse events were monitored and graded according to the Common Terminology Criteria for Adverse Events (CTCAE, version 5.0). Side effects possibly related to melatonin, including daytime sleepiness, dizziness, headache, decreased concentration, and nightmares, were recorded at each visit and systematically reported in a dedicated adverse event form. During each visit, the patients were assessed by the attending physician for clinical symptoms of heart failure.

### 3.3. Echocardiographic Data

Echocardiography was performed before initiation and one week after the last chemotherapy session. An experienced cardiologist with a fellowship in echocardiography performed all the echocardiography examinations using a Philips EPIQ CVx vendor with a phased array transducer. She was kept unaware of the course of the disease, the chemotherapy regimen, and the treatment arm. Simpson's biplane method was used to calculate volumes and LVEF. The four-chamber, three-chamber, two-chamber, and parasternal long-axis views were used to measure echocardiographic indices.

Two-dimensional speckle-tracking echocardiography was performed in three standard apical image planes using three stored image loops and auto strain LV software to measure peak longitudinal systolic strain. In this software, segmental strains were calculated using an 18-segment model. The GLS was the average segmental strain value calculated by the machine. The GLS values are reported as absolute values throughout the text. A higher GLS is indicative of a more negative GLS value.

### 3.4. Cardiac Biomarkers

The levels of cardiac biomarkers, including cTnI and CK-MB, were measured three times: Once at the initiation of chemotherapy and repeated one week after the second and one week after the fourth chemotherapy session. The level of CK-MB was measured by the International Federation of Clinical Chemistry (IFCC) method with a HITACHI 917 device produced by the Roche factory in Japan. The kit used was made by Biorex Fars Iran; up to a value of 24 IU/L is considered normal, and values more than 24 IU/L are considered indicative of a probable myocardial infarction (MI). The cTnI levels were measured by the ELFA method with the VIDAS PC device along with its special kit produced in the United States; values less than 19 ng/L were negative, 19 - 100 ng/L were borderline, and values more than 100 ng/L were considered positive.

### 3.5. Sample Size

Regarding the absence of prior human studies with melatonin in this context, the sample size was estimated based on similar studies using different drugs. The sample size was calculated using the mean ± standard deviation (SD) of LVEF reported by Hagag et al. (12) (52.25 ± 5.35 vs. 47.5 ± 5.99, P = 0.012, confidence interval: 95%, Power: 80%) and taking into account a dropout rate of 20%. The sample size was estimated to be 28 patients for each group. The aforementioned study used black seed oil against doxorubicin-induced cardiotoxicity in children with acute lymphoblastic leukemia (ALL).

### 3.6. Statistical Analysis

The normality of the continuous variables was assessed using the Shapiro-Wilk test and histogram (normal-density plot). Mean ± SD were used to describe continuous variables, and frequency and percentage were used to describe categorical variables. Comparing the mean of continuous variables between two groups was performed with Student’s *t*-test or the Mann-Whitney U test, depending on whether the variables were parametric or nonparametric. The chi-square test was used to compare the difference between the frequencies of categorical variables. To compare the paired continuous values before and after the intervention, paired *t*-tests or Wilcoxon tests were used based on the normality of the data. Effect size for GLS and LVEF changes was calculated using Cohen’s d, based on the mean difference between groups divided by the pooled SD.

Analyses were conducted according to a modified intention-to-treat (mITT) principle, defined as including all randomized participants with at least one post-baseline measurement. Repeated measures analysis of variance (ANOVA) with Bonferroni post hoc analysis was used to test changes in cardiac biomarkers between intervention groups. Sphericity assumptions were assessed using Mauchly’s test, and Greenhouse-Geisser, Huynh-Feldt, or multivariate approaches were used in appropriate situations. All statistical analyses were conducted at a significance level of less than 0.05 using STATA version 14 software. Importantly, no missing data were present, and intention-to-treat analysis was preserved.

## 4. Results

### 4.1. Patient Recruitment

**Figure 1.**
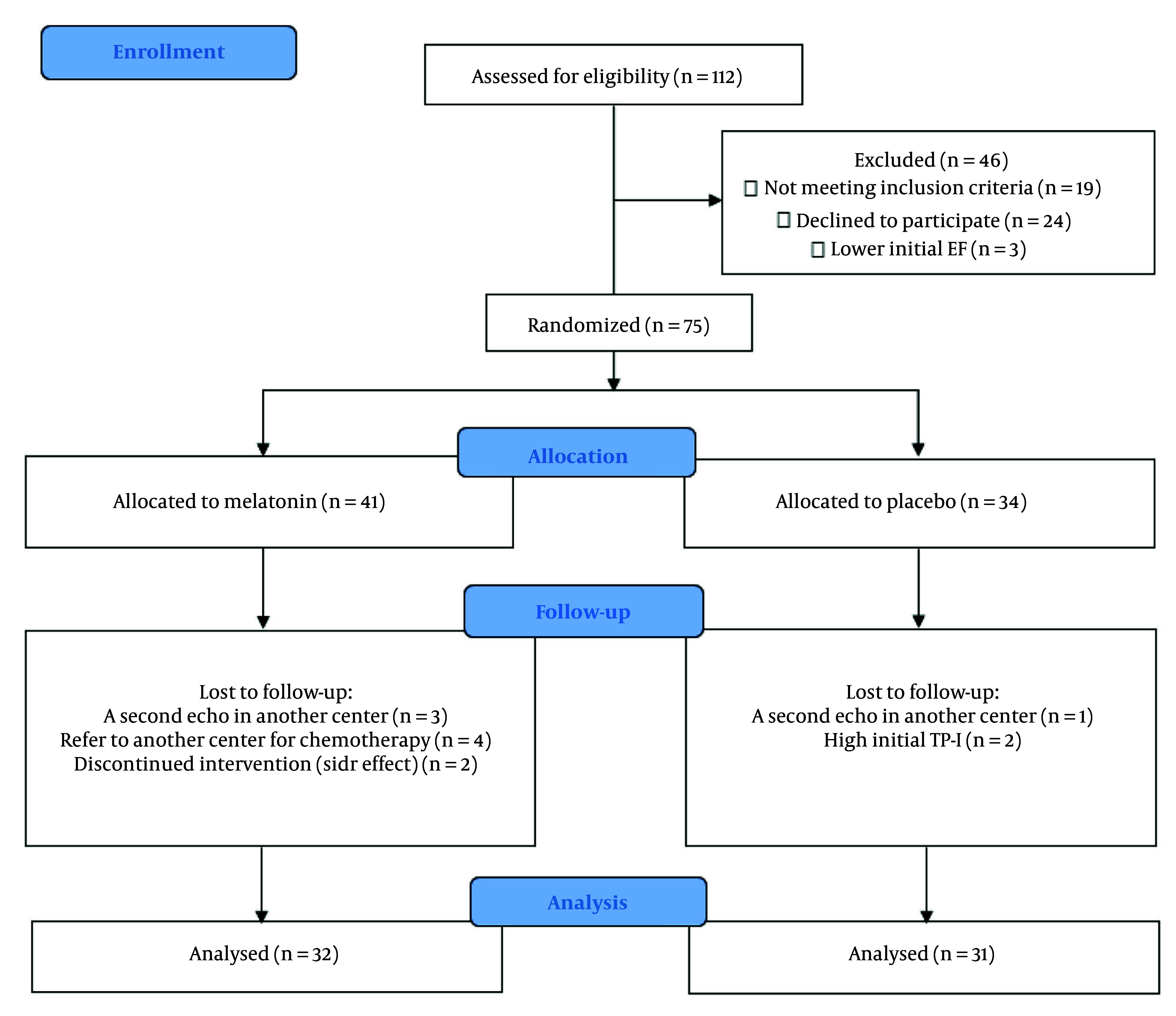
CONSORT flow diagram

From April to December 2022, 121 patients were evaluated for enrollment in this study, of whom 75 were included. Eventually, 63 patients (32 patients in the melatonin group and 31 patients in the placebo group) remained until the end of the study, and their data were analyzed (Figure 1). All patients adhered to the study medication, and two patients discontinued melatonin due to adverse effects (headache with dizziness in one case, and daytime sleepiness with dullness in another). No serious adverse drug reactions were observed.

### 4.2. Baseline Characteristics

Baseline demographic, laboratory, and echocardiographic data are shown in Table 1, indicating no significant differences between the two groups before initiating the chemotherapy agents.

**Table 1.** Comparison of Patients' Baseline General and Clinical Information Between the Two Groups ^a^

Variables	All Patients (N = 63)	Melatonin (N = 32)	Placebo (N = 31)	P-Value
**General information**				
Age (y)	44.25 ± 9.43	42.09 ± 7.08	46.48 ± 11.03	0.067
Weight (kg)	66.42 ± 9.70	67.06 ± 9.46	65.77 ± 10.05	0.602
**Chemotherapy regimen**				0.877
Two wk AC regimen	25 (39.68)	13 (40.63)	12 (38.71)	
Three wk AC regimen	38 (60.32)	19 (59.38)	19 (61.29)	
**Echocardiography’s results**				
Baseline LVEF (%)	54.60 ± 1.71	54.37 ± 2.09	54.83 ± 1.21	0.159
Baseline GLS (%) ^b^	16.53 ± 3.77	16.20 ± 3.78	16.88 ± 3.80	0.483
**Cardiac biomarkers**				
Baseline cTnI (ng/mL)	2.09 ± 0.46	2.15 ± 0.49	2.03 ± 0.42	0.203
Baseline CK-MB (IU/L)	15.39 ± 3.16	15.80 ± 3.50	14.96 ± 2.77	0.208

Abbreviations: AC, cyclophosphamide; LVEF, left ventricular ejection fraction; GLS, global longitudinal strain; cTnI, cardiac troponin I; CK-MB, creatine kinase-myoglobin binding.

^a^ Values are expressed as mean ± standard deviation (SD) or No. (%).

^b^ GLS values are reported as absolute values.

### 4.3. Echocardiography Data

According to Table 2, the average LVEF in both groups significantly decreased compared to the baseline. Moreover, there was a significant decrease (-3.68 ± 3.06) in the average GLS in the placebo group compared to the baseline (P < 0.001). In contrast, the GLS decline in the melatonin group was minimal (-0.75 ± 3.22) and was not statistically significant (P = 0.192).

**Table 2.** Changes in Left Ventricular Ejection Fraction and Global Longitudinal Strain Before and After Intervention in Each Group ^a^

Groups	First Measurement	Last Measurement	Difference of LVEF (%) or GLS (%); (Mean Difference ± Standard Deviation Difference) ^b^	P-Value (Within Group Comparison)
**LVEF**				
Melatonin (n = 32)	54.37 ± 2.09	52.78 ± 2.45	-1.59 ± 2.67	0.0006 ^c^
Placebo (n = 31)	54.83 ± 1.21	52.74 ± 1.73	-2.09 ± 1.75	< 0.001 ^c^
**GLS ** ^**d**^				
Melatonin	16.20 ± 3.78	15.44 ± 2.86	-0.75 ± 3.22	0.192
Placebo	16.88 ± 3.80	13.20 ± 2.99	-3.68 ± 3.06	< 0.001 ^c^

Abbreviations: LVEF, left ventricular ejection fraction; GLS, global longitudinal strain.

^a^ Values are expressed as mean ± standard deviation (SD).

^b^ Difference = Value after - Value before.

^c^ Statistically significant, P-value < 0.05.

^d^ The GLS values are reported as absolute values.

### 4.4. Comparison of Echocardiography Results Between Groups

According to Table 3, the reduction in LVEF between the melatonin and placebo groups and the final LVEF was not statistically significant (P = 0.110 and 0.517, respectively). On the other hand, the GLS reduction in the melatonin group was significantly lower, and the final GLS was significantly higher compared to the placebo group at the end of the study (P = 0.0005 and 0.003, respectively).

**Table 3.** Comparison of Left Ventricular Ejection Fraction and Global Longitudinal Strain Between Groups ^a^

Echocardiography Parameters	Melatonin (N = 32)	Placebo (N = 31)	Cohen’s d (Effect Size)	Between Groups Comparison ^b^
**LVEF**				
First measurement (LVEF, %)	54.37 ± 2.09	54.83 ± 1.21	-	0.159
Last measurement (LVEF, %)	52.78 ± 2.45	52.74 ± 1.73	-	0.517
1Difference of measured LVEF (%) ^c^	-1.59 ± 2.67	-2.09 ± 1.75	0.22	0.110
< 10% decrease in LVEF	31 (96.88)	31 (100.00)	-	1.00
≥ 10% decrease in LVEF	1 (3.13)	0 (0.00)	-	1.00
**GLS**				
First measurement (GLS, %)	16.20 ± 3.78	16.88 ± 3.80	-	0.483
Last measurement (GLS, %)	15.44 ± 2.86	13.20 ± 2.99	-	0.003 ^d^
Difference of measured GLS (%)	-0.75 ± 3.22	-3.68 ± 3.06	0.93	0.0005 ^d^
≤ 15% decrease in GLS	25 (78.13)	8 (25.81)	-	< 0.001 ^d^
> 15% decrease in GLS	7 (21.88)	23 (74.19)	-	< 0.001 ^d^

Abbreviations: LVEF, left ventricular ejection fraction; GLS, global longitudinal strain.

^a^ Values are expressed as mean ± standard deviation (SD).

^b^ Comparison of difference values (GLS or LVEF) between two groups of intervention.

^c^ Difference = Value last - Value first.

^d^ Statistically significant, P-value < 0.05.

According to the CTRCD definitions in the 2022 ESC Guidelines on cardio-oncology (6), none of our patients were in the severe symptomatic or severe asymptomatic CTRCD (HF) group. Thirty patients had mild asymptomatic CTRCD (defined as LVEF ≥ 50% and a new relative decline in GLS by 15% from baseline), of which 23 patients (74%) were in the placebo group and seven patients (21%) were in the melatonin group, and the difference was statistically significant (P < 0.001).

### 4.5. Cardiac Biomarkers

**Figure 2.**
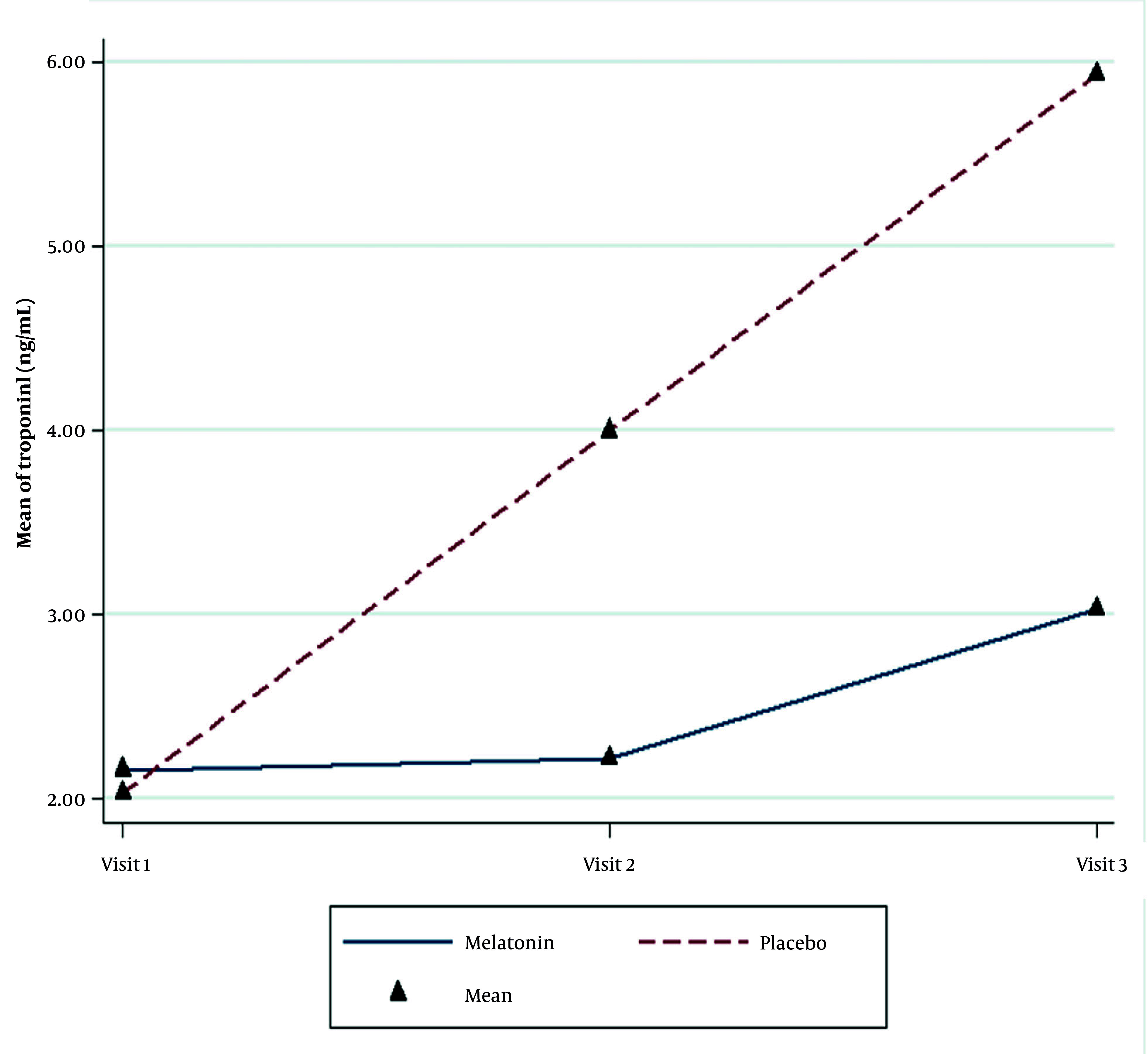
Changes in mean troponin I levels in the two groups

The average cTnI level in the second and third measurements increased significantly in both groups. Nevertheless, this increase was significantly higher in the placebo group than in the melatonin group (P < 0.001, Table 4). An increase in the cTnI levels during the study was significant in both groups, but the increment amount was more intense in the placebo group (P < 0.001, Figure 2). 

**Table 4.** Cardiac Biomarker Changes at the Three Intervention Visits Between Groups ^a^

Cardiac Biomarkers	Visit 1 (T1)	Visit 2 (T2)	Visit 3 (T3)	P-Value			Post-hoc for Time Groups ^b^
				Time Effect	Time × Groups	Between Groups	
**CTnI (ng/mL)**					< 0.001 ^c^	< 0.001 ^c^	
Melatonin	2.15 ± 0.49	2.21 ± 0.60	3.03 ± 3.90	0.032 ^c^			T1/T2 ^c^
Placebo	2.03 ± 0.42	4.00 ± 1.46	5.93 ± 2.61	< 0.001 ^c^			T1/T2 ^c^; T1/T3 ^c^; T2/T3 ^c^
**CK-MB (IU/L)**					0.006 ^c^	0.628	
Melatonin	15.80 ± 3.50	15.51 ± 3.48	15.64 ± 3.79	0.512			-
Placebo	14.96 ± 2.77	16.27 ± 2.73	17.95 ± 7.37	< 0.001 ^c^			T1/T2 ^c^; T1/T3 ^c^

Abbreviations: CTnI, cardiac troponin I; CK-MB, creatine kinase-myoglobin binding.

^a^ Values are expressed as mean ± standard deviation (SD).

^b^ Multiple comparisons were based on Bonferroni post-hoc analysis for comparing values in different visit times.

^c^ Statistically significant, P-value < 0.05 based on repeated measure analysis of variance (ANOVA).

**Figure 3.**
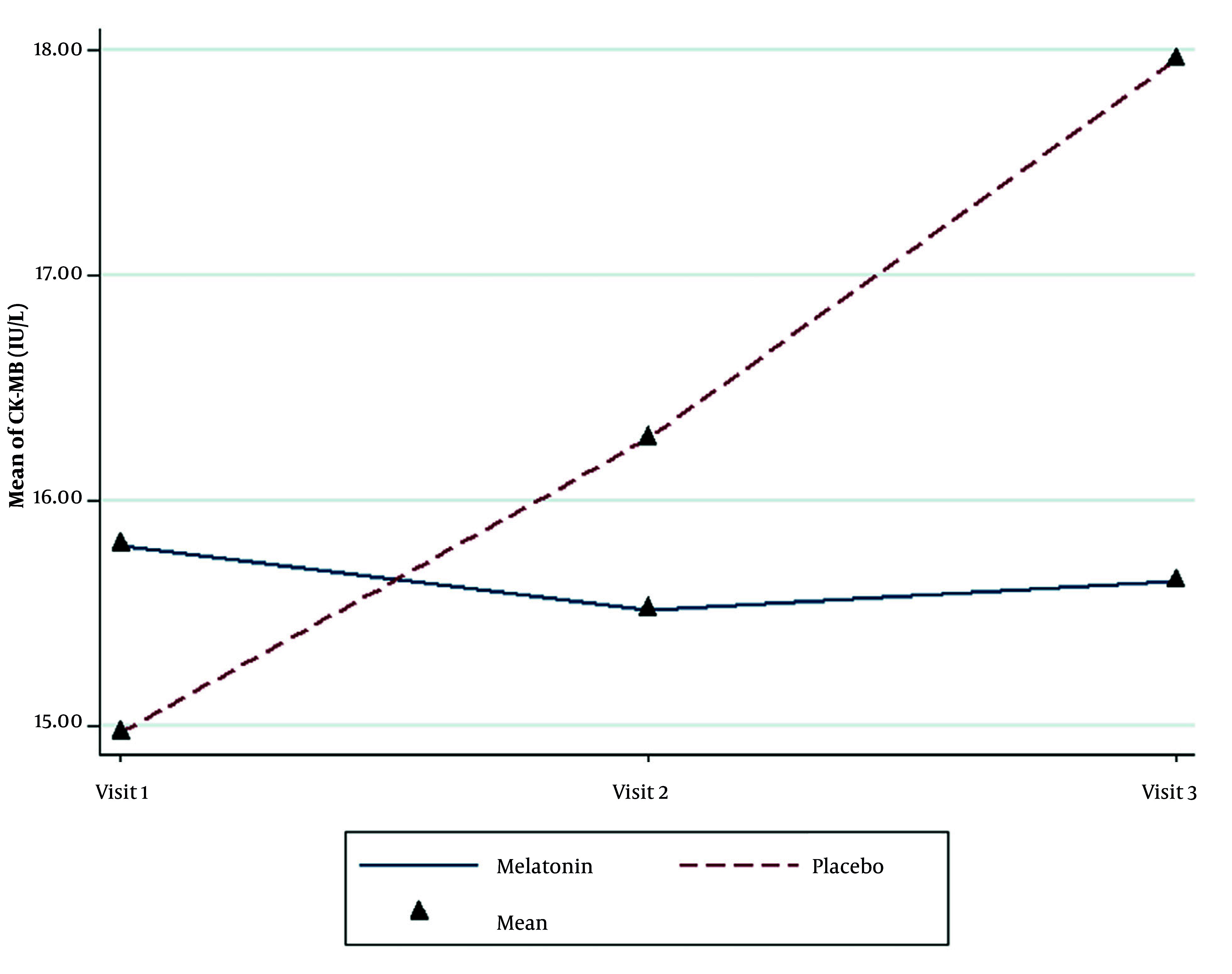
Changes in the mean creatine kinase-myoglobin binding (CK-MB) level in the two groups

The CK-MB level in the melatonin group did not change significantly during the study (P = 0.512), while a significant increase occurred in the placebo group (P < 0.001). Although the use of melatonin was significantly effective in preventing the rise in CK-MB levels (P = 0.006), the differences in CK-MB between the two groups were not significant (P = 0.628, Figure 3 and Table 4). 

## 5. Discussion

This randomized, placebo-controlled clinical trial investigated the efficacy of melatonin in the prevention of doxorubicin-induced cardiotoxicity. Melatonin administration was associated with the preservation of GLS and lower increases in cardiac biomarkers (cTnI and CK-MB), while LVEF changes did not differ significantly between groups. To our knowledge, this is the first human study investigating the role of melatonin in the prevention of chemotherapy-induced cardiotoxicity. Melatonin, as an antioxidant agent, has been studied in various in vitro studies to reduce the cardio-cytotoxic effects of doxorubicin (19, 21, 22). This emphasizes the novelty of our trial as the first randomized controlled trial in humans evaluating melatonin for anthracycline-induced cardiotoxicity.

The primary mechanism of cardiotoxicity by anthracyclines is based on oxidative stress caused by the production of oxygen free radicals during lipid peroxidation of cell membranes and mitochondria. In addition, other mechanisms include the reduction of myocardial cells due to damage to cell membranes and mitochondria, calcium imbalance, and apoptosis (12, 23). Exogenous antioxidants have been reported in many in vitro and in vivo studies to reduce this complication (12, 16, 22, 24). Consistent with this, recent preclinical studies have demonstrated that melatonin can attenuate the cardiotoxic effects of doxorubicin by mitigating its inflammatory and oxidative consequences (7, 15-17). Melatonin has many advantages, including no adverse effects on hemodynamics with no bradycardia or blood pressure drop, and it strengthens the antitumor effects of anthracyclines (17, 25). Moreover, the low cost and wide availability of the drug make it an exciting option to assess in this clinical research.

In this study, echocardiography follow-ups were accomplished by measuring 2D LVEF using Simpson’s method and GLS using 2D speckle echocardiography. After chemotherapy, the average LVEF decreased in both groups, with a slightly greater decrease in the placebo group, but the difference between the two groups remained nonsignificant (P = 0.110). Conversely, the average GLS did not change significantly in the melatonin group (P = 0.192), while the patients in the placebo group experienced a significant (P < 0.001) decrease in average GLS. At the end of the study, the difference in GLS between the two groups was also significant (P = 0.0005).

Echocardiography has been the main method for assessing cardiac health in cancer patients, with LVEF being the primary parameter examined in the context of cardiotoxicity (26). Considering the irreversible nature of anthracycline cardiotoxicity, early diagnosis of subclinical involvement is paramount. Early detection of asymptomatic cardiotoxicity leads to faster initiation of treatment, which can prevent worsening cardiac involvement and increase survival. Therefore, in recent years, many studies have supported using GLS and measuring the level of cardiac biomarkers for this purpose (9, 26-28). The European Society of Cardiology's latest guidelines on cardio-oncology emphasize the importance of GLS measurement in assessing the cardiotoxicity of cancer treatments. A decrease in GLS of more than 15% during treatment is the recommended threshold for suspecting subclinical cardiac dysfunction (6). Abnormal GLS can predict future LVEF reduction (29). Its advantage over LVEF is that it is less affected by the physiological conditions of the person; it is more sensitive and can help in the early diagnosis of subclinical cardiotoxicity (30, 31). According to a systematic review, early deterioration of GLS can predict LVEF decline three months later (32).

In parallel with the results of our study, Livi et al. as cited by Omland et al. discovered that breast cancer patients taking an anthracycline experienced a lesser decrease in GLS when given cardioprotective drugs such as ramipril and bisoprolol, compared to the placebo group. This finding supports the use of GLS as a highly sensitive tool for diagnosing subclinical cardiotoxicity (33). Additionally, van der Linde et al. demonstrated that GLS can identify indications of LV dysfunction prior to a decrease in 3D LVEF measurement (29). Considering the results of our study, melatonin, by preventing a GLS drop, may help prevent cardiotoxicity caused by doxorubicin.

Previous studies reported that doxorubicin, by damaging cardiac cells, leads to increased cardiac injury markers, including troponin I and CK-MB (21, 34). Some clinical studies have shown that elevated troponin levels during chemotherapy are an early marker of an increased risk of left ventricular dysfunction (9, 35). Similarly, in many animal studies, melatonin administration with doxorubicin decreased cardiac biomarkers, including troponin and CK-MB (21, 34, 36). In a review article conducted by Ananthan and Lyon, the importance of monitoring troponin levels during anthracycline treatment was highlighted, as it is a reliable indicator of cardiac dysfunction (28). A randomized controlled trial demonstrated that melatonin significantly reduced CK-MB levels after percutaneous coronary intervention, supporting its cardioprotective effect (37). In this context, our data also showed that the increase in the level of cardiac biomarkers such as cTnI and CK-MB was lower in the melatonin group than in the placebo group, which confirms the role of melatonin in controlling and inhibiting cardiotoxicity caused by doxorubicin.

**Figure 4.**
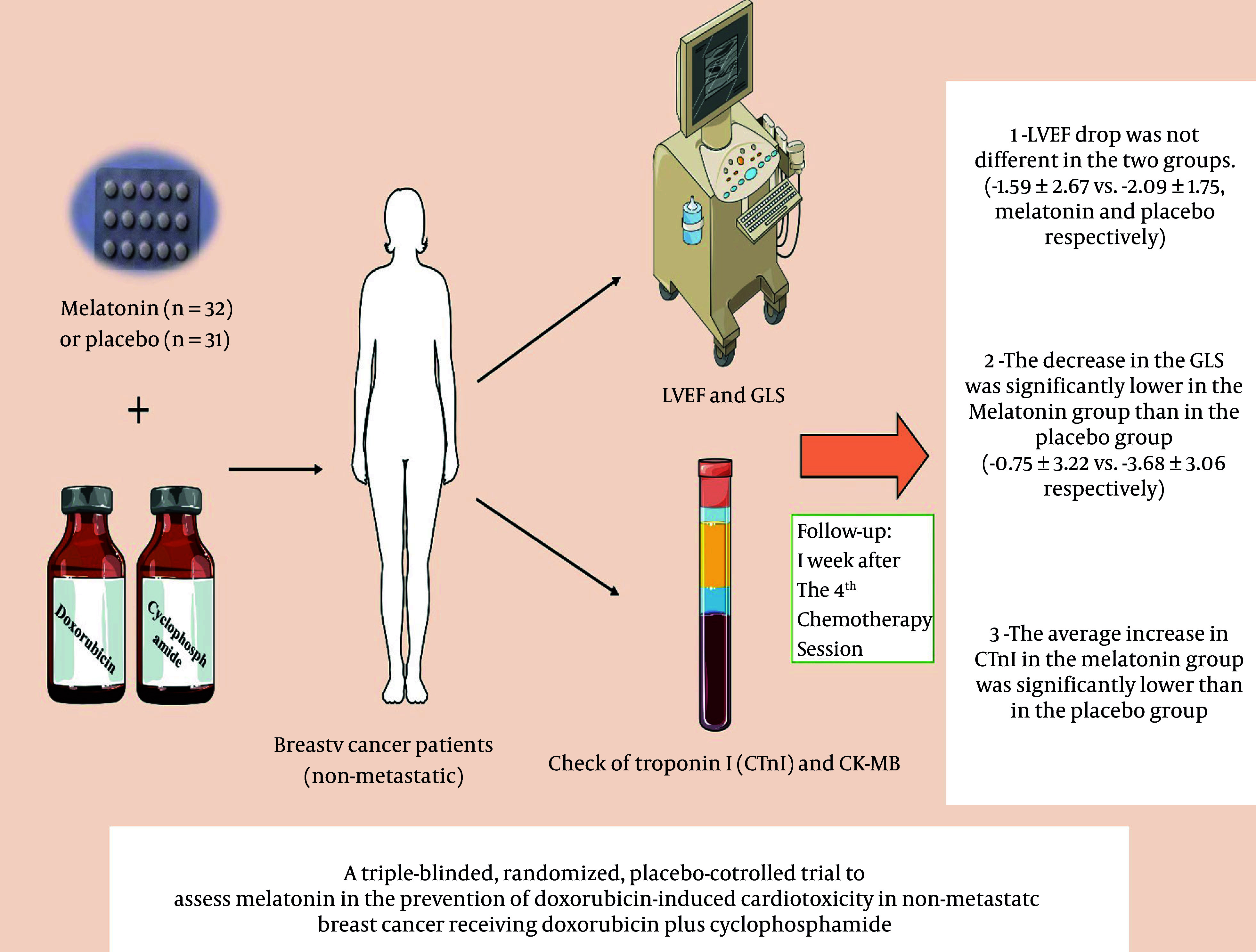
Central illustration: A summary of the obtained results from the administration of Melatonin as a combination therapy with doxorubicin to reduce cardiotoxicity caused by doxorubicin in patients with newly diagnosed non-metastatic breast cancer receiving a first session of AC chemotherapy regimen. Triple-blind clinical trial in the Cancer Institute Center of Imam Khomeini Hospital Complex, Tehran University of Medical Sciences.

Overall, the results of our study showed that the administration of melatonin may be effective in reducing subclinical cardiac toxicity caused by doxorubicin due to its low cost, low complications, and availability, making it a very suitable option, especially in countries with lower income (Figure 4). 

### 5.1. Conclusions

In conclusion, the results of the present study showed that melatonin may help reduce the subclinical cardiotoxicity caused by doxorubicin in breast cancer patients. Prophylactic use of melatonin could be effectively confirmed by preventing the decrease in GLS and the increase in cardiac biomarkers (cTnI and CK-MB) in patients receiving doxorubicin. Although many in vitro studies have been conducted on the mechanism of melatonin's effect in reducing cardiotoxicity caused by doxorubicin consumption, the present study is the first clinical trial in this context. Toward this end, it is suggested to conduct larger multicenter clinical studies to confirm the data of the present study.

### 5.2. Study Limitations

The principal limitation of this study was the relatively short follow-up period, which restricts the assessment of the sustained effects of melatonin. Furthermore, high-risk populations such as patients with diabetes were excluded; thus, generalizability to broader populations may be limited. Adherence monitoring was based on self-reporting and blister return, which may be prone to reporting bias. Additionally, measurement of NT-proBNP, a valuable biomarker for cardiotoxicity, was not feasible due to its high cost.

## Data Availability

The dataset presented in the study is available on request from the corresponding author during submission or after publication. The data are not publicly available due to privacy and ethics.

## Clinical Trial Registration Code:

IRCT20211109053025N1.
